# Network Meta-Analysis of the Effectiveness of Neoadjuvant Endocrine Therapy for Postmenopausal, HR–Positive Breast Cancer

**DOI:** 10.1038/srep25615

**Published:** 2016-05-13

**Authors:** Wei Wang, Chenghao Liu, Wenbin Zhou, Tiansong Xia, Hui Xie, Shui Wang

**Affiliations:** 1 Department of Breast Surgery, The First Affiliated Hospital with Nanjing Medical University, 300 Guangzhou Road, Nanjing 210029, China

## Abstract

In clinical practice, it is necessary to define an optimal choice from many different therapeutic regimens. This study aimed to assess the efficacy and safety of neoadjuvant endocrine therapy (NET) for breast cancer patients. Randomized clinical trials were included. Nine studies comprising 2133 patients were included in the final analysis. Network meta-analysis showed that everolimus plus letrozole was more easily accepted by patients than exemestane (≥20wks) (odds ratio (OR): 856697.02, 95% confidence intervals (95%CI): 1.88 to 87242934...); exemestane (≥20wks) had worse acceptability than letrozole (OR: 0.00, 95%CI: 0.00 to 0.98). Letrozole produced a better clinical objective response (COR) than tamoxifen (OR: 1.99, 95%CI: 1.04 to 3.80). The incidence of fatigue between the anastrozole plus gefitinib group and the everolimus plus letrozole group was significantly different (OR: 0.08, 95%CI: 0.01 to 0.83). The exemestane (<20wks) plus celecoxib group had fewer hot flushes than others. Ranking showed the everolimus plus letrozole was most likely rank first in comparisons of COR and acceptability, and had a 64% possibility to rank first after stochastic multi-criteria acceptability analysis. In conclusion, our study showed that letrozole plus everolimus is the most effective treatment for postmenopausal, hormone receptor-positive breast cancer in the neoadjuvant setting.

Breast cancer is a common malignant disease worldwide. Surgery, systemic therapy and radiotherapy, as the main treatment modalities, have significantly improved the prognosis of breast cancer^1^. Neoadjuvant endocrine therapy (NET), with the advantage of downsizing the tumor before surgery, provides a therapeutic alternative for patients with hormone receptor-positive (HR-positive), postmenopausal breast cancer^2^. Recently, many randomized clinical trials (RCTs) concerning NET have emerged and its clinical application is gradually gaining recognition. Based on the available research conclusions, more than 90% of experts voted for the use of NET in patients with HR-positive breast cancer during the 13th St. Gallen International Breast Cancer Conference^3^. Although some research results for NET have been reported, it is difficult to integrate information on the relative efficacy of all tested regimens because most individual trial compared only a few treatments; it is impossible to involve all therapeutic regimens in one trial^4^. Thus, a summary of these trials may be needed. Network meta-analysis not only synthesizes information from different trials and combines direct and indirect evidence on the relative effectiveness of the treatments, but also can tell us which regimen is appropriate after comparisons of the benefits and risks based on the evidence^5^,^6^.

In this study, we assessed the efficacy and safety of NET systematically for postmenopausal, HR-positive, non-metastatic breast cancer by conducting direct and indirect comparisons from RCTs. We aimed to provide a useful summary of different treatment regimens that could be used to guide treatment decisions.

## Results

### Overview of the Literature Search and Study Characteristics

**Figure 1:**
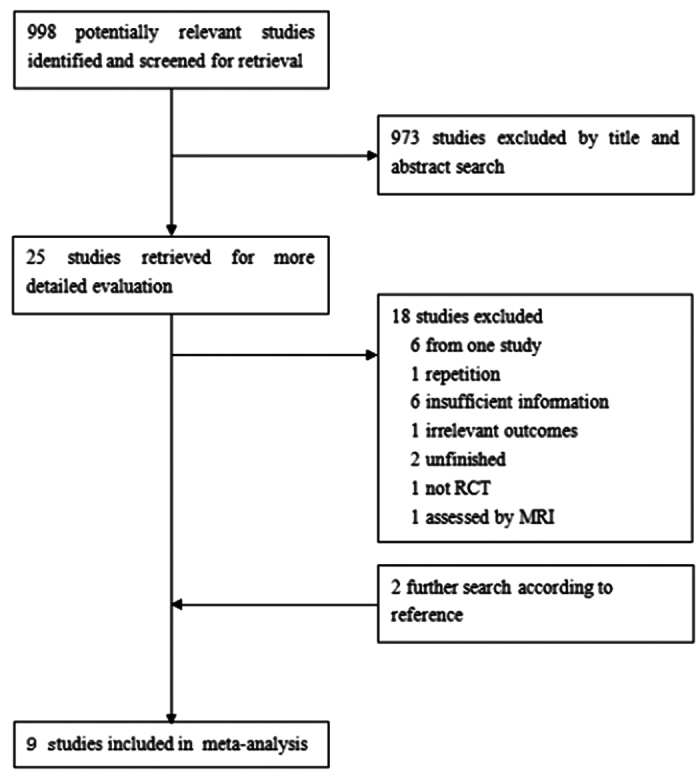
Consort diagram of study selection.

A total of 998 articles were identified in the original database search, of which 973 were discarded after reviewing the titles and abstracts because they clearly did not meet the criteria for inclusion. The remaining full texts were read and six papers were excluded because they derived from two trials. Two papers were repetitive and one was reserved. Another eleven studies were discarded because six studies provided results from either a too small sample size or obviously inadequate information; the tumor size in one study was not assessed using calipers; only therapeutic effects of different dose of fulvestrant were reported in one study; two studies are still under way; and the last one was not a randomized trial. Finally, nine studies were identified and included (Fig. 1)^7^,^8^,^9^,^10^,^11^,^12^,^13^,^14^,^15^.

### The Assessment of the Risk of Bias

The pooled risks of bias for the different studies included in this network analysis are presented in Supplementary Figure 1.

### Results of Direct Comparisons

The nine studies comprised 2133 patients. The duration of treatment was from 12 to 24 weeks. An investigation into the optimal duration of exemestane was reported in one study^8^. To make a distinction, we defined exemestane (<20wks) if the duration of exemestane was less than 20 weeks, and exemestane (≥20wks) if the treatment duration was than 20 weeks. There were three arms in two studies, respectively. One study was about anastrozole plus different treatment protocols of gefitinib compared with anastrozole, and we considered anastrozole versus anatrozole plus gifitinib^12^. As a result, ten arms were assessed including, chemotherapy, tamoxifen, letrozole, anastrozole, exemestane (≥20wks), exemestane (<20wks), anastrozole plus tamxifen, letrozole plus everomilus, anatrozole plus gefitinib, and exemestane (<20wks) plus celecoxib. All patients were postmenopausal women diagnosed with non-metastatic breast cancer. All patients except for four were HR-positive^15^. Four studies reported the levels of HER2^7^,^8^,^9^,^11^. Characteristics of the eligible studies are listed in Table 1.

**Figure 2:**
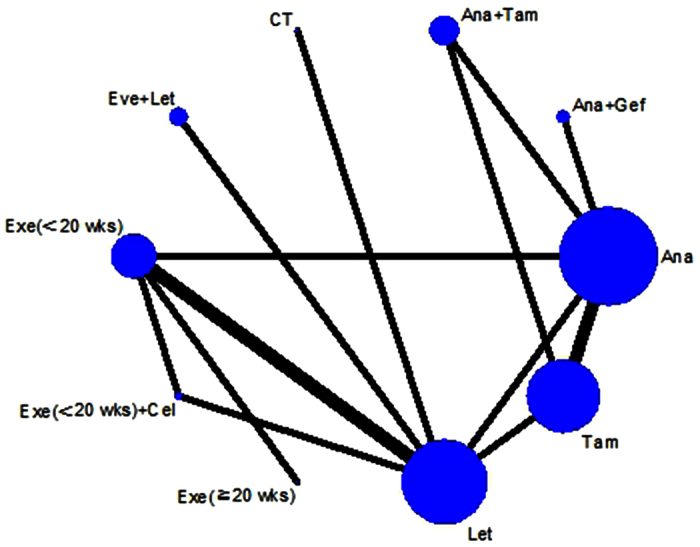
Network diagram of studies comparing clinical objective response (COR) of different neoadjuvant endocrine therapy (NET) therapies for HR-positive breast cancer. Each link represents at least 1 study and the widths of each link are proportional to the number of studies comparing the particular arms. The size of each node is proportional to the total sample size. CT = chemotherapy, Ana = anastrozole, Tam = tamoxifen, Gef = gefitinib, Let = letrozole, Exe (<20wks) = Exemestane (<20wks), Exe (≥20wks) = Exemestane ((≥20wks), Cel = Celecoxib, Eve = Everolimus.

The numbers of patients who achieved a clinical objective response (COR) and completed treatment were reported in nine studies. Eight studies provided information about fatigue and hot flushes, and seven studies reported the number of patients that received breast conserving surgery (BCS) after NET. Pathological complete response (pCR) was reported in four studies and only eight (1.1%) patients achieved pCR^8^,^11^,^15^. Direct comparisons were performed and are listed in Table 2. Forest plots are shown in Supplementary Figures 2–6. From the eligible studies, a network diagram of the studies comparing COR was done using Stata, and the result are shown in Fig. 2.

From direct comparisons, we found that the COR rate in the letrozole group was significantly higher than that in the tamoxifen group (odds ratio (OR): 2.20, 95% confidence interval (95%CI): 1.41 to 3.44, p = 0.001) or the exemestane (<20wks) group (OR: 1.63, 95%CI: 1.01 to 2.64, p = 0.042). Significantly worse acceptability of letrozole was observed compared with letrozole plus everolimus (OR: 0.5, 95%CI: 0.28 to 0.87, p = 0.015); however, the incidence of fatigue in the letrozole group was remarkably lower than in the letrozole plus everolimus group (OR: 0.34, 95%CI: 0.13 to 0.89, p = 0.028). Besides, patients taking anastrozole suffered less fatigue than those taking tamoxifen (OR: 0.47, 95%CI: 0.22 to 0.98, p = 0.044). The incidence of hot flushes in the letrozole group was significantly higher than in the exemestane (<20wks) (OR: 2.47, 95%CI: 1.30 to 4.70, p = 0.006), exemestane (<20wks) plus celecoxib (OR: 8.44, 95%CI: 2.55 to 27.91, p = 0.0001) or chemotherapy (OR: 6.92, 95%CI: 1.29 to 37.29, p = 0.024) groups. More patients accepted BCS after taking anastrozole than among those taking tamoxifen (OR: 1.95, 95%CI: 1.26 to 3.02, p = 0.003) or letrozole (letrozole *vs*. anastrozole (OR: 0.39, 95%CI: 0.18 to 0.84, p = 0.016)).

### Bayesian Network Meta-Analysis

To assess the consistency and inconsistency in the network meta-analysis, node-splitting analyses were performed. Which revealed no statistical differences between the direct and indirect evidence. From the eligible studies, indirect comparisons were then performed. The outcomes of indirect comparisons of COR, treatment completion (TC) and adverse events are shown in Tables 3 and 4.

Network meta-analysis showed that everolimus plus letrozole more easily accepted by patients than exemestane (≥20wks) (OR: 856697.02, 95%CI: 1.88 to 87242934…), and exemestane (≥20wks) was also had worse acceptability than letrozole (OR: 0.00, 95%CI: 0.00 to 0.98). There was a statistically significant difference between letrozole and tamoxifen group in the comparison of COR (OR: 1.99, 95%CI: 1.04 to 3.80). In addition, the incidence of fatigue between the anastrozole plus gefitinib group and the everolimus plus letrozole group showed a significant difference (OR: 0.08, 95%CI: 0.01 to 0.83). The incidence of hot flushes in the exemestane (<20wks) plus celecoxib group seem to be the lowest and four comparisons had statistically significant differences: anastrozole *vs*. exemestane (<20wks) + celecoxib (OR: 8.44, 95%CI: 1.53 to 48.18), anastrozole + tamoxifen *vs*. exemestane (<20wks) + celecoxib (OR: 13.11, 95%CI: 1.76 to 109.65), exemestane (<20wks) + celecoxib *vs*. letrozole (OR: 0.11, 95%CI: 0.02 to 0.47), and exemestane (>20wks) + celecoxib *vs*. tamoxifen (OR: 0.99, 95%CI: 0.02 to 0.52)). Furthermore, the incidence of hot flushes in the chemotherapy group was significantly lower than in another three treatment regimens (chemotherapy *vs*. letrozole (OR: 0.12, 95%CI: 0.01 to 0.76), chemotherapy *vs*. tamoxifen (OR: 0.11, 95%CI: 0.01 to 0.86), and anastrozole plus tamoxifen *vs*. chemotherapy (OR: 11.94, 95%CI: 1.14 to 171.80)).

Rankings for the outcomes of COR, TC, BCS and adverse events in the present analysis were also performed. The probabilities were calculated for a total of 100%, both within a rank over interventions and within an intervention over ranks. The top and second highest percentage within each intervention are shown in Table 5. Besides, a subgroup analysis was performed involving complete response (CR) and partial response (PR) (Supplementary Figures 7–13).

Rankings showed that everolimus plus letrozole had the highest probability to rank first in the comparisons of COR (62%), PR (45%) and acceptability (44%). Seven studies reported information about the BCS rate. From the limited data, we found that more patients could accept BCS after receiving anastrozole plus gefitinib (69%).

### Stochastic Multi-criteria Acceptability Analysis (SMAA)

The SMAA benefit-risk analyses were based on evidence synthesis. The criteria were COR, TC, and the alternatives were treatment arms. Ranking for SMAA benefit-risk analysis is shown in Supplementary Figure 14. The first and second high percentages within each intervention over ranks are shown in Table 5.

SMAA benefit-risk analyses suggested that everolimus plus letrozole, having a 64% possibility to rank first, was the best treatment arm when considering COR and TC, and letrozole was the second choice.

## Discussion

The use of neoadjuvant chemotherapy in the treatment of locally advanced breast cancer is well established. However, endocrine therapy, with lower toxicity, can be a valid alternative to chemotherapy in the treatment of hormone-sensitive tumors, particularly in postmenopausal women^16^. It can downsize tumors and provide an early measurement tool to evaluate response to endocrine therapy^3^. Here, we presented a meta-analysis of the efficacy of the available studies involving NET.

From the direct and indirect comparisons, we found that the letrozole group had a higher COR rate than the tamoxifen group. This was consistent with previous reports. For example, a study involving meta-analyses of two cohorts concerning adjuvant endocrine therapy demonstrated efficacy and superiority of aromatase inhibitors (AIs) when compared with tamoxifen^17^,^18^. In breast cancer, the PI3K/Ak/mTOR pathway is important in the clinical sensitivity of breast cancer to endocrine therapy. Everolimus, an mTOR inhibitor, can restore sensitivity to endocrine therapy^19^. A phase III randomized trial showed that everolimus combined with an AI could improve progression-free survival in patients with HR-positive, advanced breast cancer previously treated with non-steroidal AIs^20^. Ranking in this study also showed that everolimus plus letrozole might be the best choice for patients to reach COR and was more easily accepted.

In addition, we found that chemotherapy was the first choice for patients to obtain a CR. However, a phase 2 randomized trial of primary endocrine therapy versus chemotherapy did not show a significant difference for pCR (3% *vs*. 6%) and disease progression (9% *vs*. 9%) rates, respectively (p>0.05). Besides, the rate of BCS was slightly higher in the endocrine group (33% *vs*. 24%; p = 0.058)^21^. The sample size in the chemotherapy arm was small and pCR was not analyzed in this study; therefore, more trials will be needed to compare NET with chemotherapy.

Compared with chemotherapy, an important superiority of endocrine therapy is its lower toxicity. In the nine included studies, severe adverse events were rarely reported and the most common side effects were fatigue and hot flushes. Although everolimus plus letrozole produced a higher incidence of fatigue, it was still more easily accepted. Ranking also showed that everolimus plus letrozole had the highest probability to rank first for acceptance. Hot flushes were another common side effect. This study suggested that anastrozole plus tamoxifen had a 57% probability to rank first and exemestane (<20wks) plus celecoxib had a 47% probability to rank last. Therefore, when patients have severe hot flushes after receiving endocrine therapy, celecoxib, a non-steroidal anti-inflammatory drug (NSAID), is a good choice.

In this study, there was no significant difference between the exemestane (≥20wks) and exemestane (<20wks) groups in terms of reaching COR and complete treatment. Ranking also struggled to decide which one was better than the other. In addition, adverse events in the original study investigating optimal duration of exemestane therapy were not available^8^. Thus, it was hard to produce a comprehensive analysis. SMAA suggested that exemestane (≥20wks) ranked last and exemestane (<20wks) ranked sixth or seventh. A phase II study that investigated preoperative treatment with exemestane for 6 months in postmenopausal patients with HR-positive breast cancer showed a more beneficial effect for 6 months^22^. However, Hojo demonstrated that responses were equal during 4 or 6 months of exemestane treatment, and showed that 4-months of treatment with exemestane appeared to be warranted in postmenopausal patients because of its increased acceptability^8^. Therefore, the optimal duration of exemestane remains controversial.

This study provided an insight into the NET for HR-positive, postmenopausal breast cancer. However, it had some limitations. First, the number of studies and the patients included are relatively limited. Second, for the comparisons in the network meta-analysis, no direct evidence was available, and indirect comparisons might cause heterogeneity. Third, we did not consider the influence of diversity of ethnicity and the SMAA benefit-risk analysis only analyzed two criteria^23^. Finally, the indicator of this study is limited. The COR was restricted to being assessed using calipers, without considering other assessment methods, such as ultrasound and mammography. Therefore, future studies will be needed to assess more indicators and consider more influencing factors.

In conclusion, our study proved that letrozole plus everolimus is the most effective treatment for postmenopausal, HR-positive breast cancer in the neoadjuvant setting. In addition, when patients have hot flushes during the period of NET, NSAIDs, such as celecoxib, are recommended.

## Methods

### Search Strategy

Studies were identified by searching Embase database, the Cochrane library and PubMed with the following search terms: breast cancer or breast neoplasm or breast carcinoma; neoadjuvant or preoperative; endocrine therapy or hormonal therapy. The searches were limited to studies written in English with full text. There was no date restriction. In addition, we screened the references of all studies fulfilling the eligibility criteria in case we missed some relevant articles by the electronic searches.

### Selection Criteria

Randomized trials that compared at least two arms of different treatment regimens involving NET in postmenopausal patients with HR-positive, non-metastatic breast cancer were considered. There were no dose and duration restrictions. All titles and abstracts were screened to exclude obviously unmatched articles and the remaining full texts were read for further identification. If multiple publications of the same trial were retrieved, only the most informative publication was included. Risk of bias in the studies was assessed by two authors (Wang and Zhou) for quality; appropriateness of allocation, blinding, and management of incomplete outcome data; the completeness of reporting of outcomes and other bias using the Cochrane Collaboration risk of bias tool^24^.

### Data Extraction

A data extraction sheet based on Excel was developed. Data were extracted independently by two authors (Wang and Zhou) including: characteristics of trial participants (age, gender, menopausal status, HR and HER2 status, histological type, clinical tumor status, tumor grade and nodal status), the inclusion and exclusion criteria in each trial, type of intervention (type, dose, duration and frequency) and outcomes.

### Definition of Outcomes

The primary outcome in this study was the number of patients that achieved COR. COR included CR and PR. They were defined according to UICC, WHO or RECIST criteria. The tool used for tumor assessment in the studies was restricted to calipers. Other endpoints were the number of patients who completed treatment and the number of patients with adverse events. Adverse events were graded according to the National Cancer Institute Common Toxicity Criteria (Version 2.0 or 3.0) with no grade restrictions. The adverse events concerned in this study were fatigue and hot flushes. The numbers of patients who reached pCR and received BCS were also considered.

### Statistical Methods

In the direct comparisons, OR was utilized for pooling effect sizes because most of the outcomes were dichotomous variables. If a direct comparison was based on two or more studies, statistical heterogeneity was calculated using the *I*^*2*^ statistic. Furthermore, we defined *I*^*2*^ above 50% as a large between-study heterogeneity. If there was no significant heterogeneity, data were pooled using the Mantel-Haenszel fixed effects model^25^. Results were reported with OR and 95%CI. All statistical tests were two-sided.

For comparisons between two interventions with both direct and indirect evidence, the consistency between these types of evidence was verified by the node-split analysis provided in the Aggregate Data Drug Information System (ADDIS), an open source evidence-based drug oriented strategy decision support system^26^. If there was no significant inconsistency, the relative effects of the interventions were analyzed using a consistency model based on a random-effects Bayesian model provided by the ADDIS software^27^,^28^. Benefit-risk analysis was performed using SMAA. The results of the analysis are presented as OR with 95% CI. Ranking for each treatment was performed by calculating the probability of each arm to achieve the best rank among all treatments. In addition, sensitivity analyses were considered.

Direct comparisons and risk of bias across studies were assessment by Stata, Version 11.2 (Stata Corp, College Station, TX, USA). Risk of bias in individual studies was assessed by Review Manager (RevMan), Version 5.3 (The Nordic Cochrane Centre: The Cochrane Collaboration, Copenhagen, Norway). Bayesian network meta-analyses and the node-splitting analyses were calculated by ADDIS,Version1.16.5. The reporting of this meta-analysis was done according to Preferred Reporting Items for Systematic Reviews and Meta-Analyses (PRISMA) guidelines^29^.

## Additional Information

**How to cite this article**: Wang, W. *et al*. Network Meta-Analysis of the Effectiveness of Neoadjuvant Endocrine Therapy for postmenopausal, HR-Positive Breast Cancer. *Sci. Rep.* **6**, 25615; doi: 10.1038/srep25615 (2016).

## Supplementary Material

Supplementary Information

## Figures and Tables

**Table 1:** Characteristics of the eligible studies.

Author	Year	Country	Clinical stage	Arm	Duration (wks)	Num	Age (years), median (rang)	Postmenopausal (n)	HR+/HER2+(n)	Tumor grade (n)				Clinical tumor status (n)			Nodal status (n)		
										1	2	3	miss	T0-T2	T3	T4	N0	N1	N2-NX
C.Palmieri	2014	UK	T2/ above/any T with nodal ≥ 20mm	CT	18	22	–	22	22/2	0	15	4	3	–	–	–	–	–	–
				Let	18–23	22	–	22	22/2	1	14	4	3	–	–	–	–	–	–
Takashi Hojo	2013	Japan	IIA-IIIA	Exe (<20wks)	16	26	66 (51–80)	26	26/1	–	–	–	–	24	2	0	21	5	0
				Exe (≥20wks)	24	26	64 (57–80)	26	26/3	–	–	–	–	24	2	0	24	2	0
Matthew J.Ellis	2011	US	II or III	Exe (<20wks)	16–18	124	69 (43–90)	124	124/8	35	69	20	0	90	25	9	96	26	2
				Let	16–18	127	65 (49–90)	127	127/13	26	83	1	17	95	24	8	80	41	6
				Ana	16–18	123	65 51–87)	123	123/12	30	73	19	1	94	24	6	01	29	3
Jose´ Baselga	2009	Spain	M0	Eve+Let	16	138	69 (46–88)	138	138/–	10	50	32	46	100	29	9	84	38	9
				Let	16	132	67 (43–84)	132	132/–	8	55	20	49	102	20	10	84	36	6
Louis Wing-Cheong Chow	2008	China	NA	Exe (<20wks)+Cel	12	30	69 (49–87)	30	30/6	–	–	–	–	–	–	–	–	–	–
				Exe (<20wks)	12	24	67 (48–91)	24	24/2	–	–	–	–	–	–	–	–	–	–
				Let	12	28	75 (49–93)	28	28/2	–	–	–	–	–	–	–	–	–	–
Ian E. Smith	2007	UK	I-IIIB	Ana+Gef	16	121	–	121	121/–	23	55	14	29	110	11	77	39	5	
				Ana	16	85	70.3	85	85/–	18	33	16	18	79	6	49	35	1	
Luigi Cataliotti	2006	Italy	LABC	Ana	12	228	48.7–91.5	228	228/–	–	–	–	–	–	–	–	–	–	–
				Tam	12	223	44.1–95.9	223	223/–	–	–	–	–	–	–	–	–	–	–
Ian E. Smith	2005	UK	LABC	Ana	12	113	73.2(51.8–90.2)	113	113/–	–	–	–	–	–	–	–	–	–	–
				Tam	12	108	71.5(49.8–88.4)	108	108/–	–	–	–	–	–	–	–	–	–	–
				Ana+Tam	12	109	73.2(51.6–85.7)	109	109/–	–	–	–	–	–	–	–	–	–	–
W.Eiermann	2001	Germany	T2-T4c.N0-N2	Let	16	154	68	154	154/–		–	–	–	77	42	35	75	68	11
				Tam	16	170	67	170	166/–	–	–	–	–	91	30	49	83	66	21

CT = chemotherapy, Ana = anastrozole, Tam = tamoxifen, Gef = gefitinib, Let = letrozole, Exe (<20wks) = Exemestane (<20wks), Exe (≥20wks)= Exemestane (≥20wks), Cel = Celecoxib, Eve = Everolimus.

**Table 2:** Direct comparison for COR, TC, fatigue, hot flush and BCS.

	**Outcome**	**Events**	**Total**	**Events**	**Total**	**OR (95% CI)**
Let *vs*. Exe (<20wks)	COR	112	155	91	148	**1.63 (1.01 to 2.64)**
	TC	149	155	136	148	2.09 (0.79 to 5.54)
	Fatigue	14	155	11	148	1.23 (0.54 to 2.81)
	Hot flush	43	155	22	148	**2.47 (1.30 to 4.70)**
	BCS	11	127	17	124	0.60 (0.27 to 1.33)
Let *vs*. Exe (<20wks)+Cel	COR	17	28	18	30	1.03 (0.36 to 2.95)
	TC	28	28	29	30	2.90 (0.11 to 74.13)
	Fatigue	4	28	3	30	1.50 (0.30 to 7.39)
	Hot flush	19	28	6	30	**8.44 (2.55 to 27.91)**
	BCS	–	–	–	–	–
Let *vs*. Let+Eve	COR	78	132	94	138	0.68 (0.41 to 1.11)
	TC	90	132	112	138	**0.50 (0.28 to 0.87)**
	Fatigue	6	132	17	138	**0.34 (0.13 to 0.89)**
	Hot flush	22	132	15	138	1.64 (0.81 to 3.32)
	BCS	–	–	–	–	–
Let *vs*. CT	COR	20	22	17	22	2.94 (0.50 to 17.14)
	TC	20	22	20	22	1.00 (0.13 to 7.81)
	Fatigue	10	22	7	22	1.79 (0.52 to 6.10)
	Hot flush	9	22	2	22	**6.92 (1.29 to 37.29)**
	BCS	4	22	1	22	4.67 (0.48 to 45.62)
Let *vs*. Tam	COR	85	154	61	170	**2.20 (1.41 to 3.44)**
	TC	153	154	169	170	0.91 (0.06 to 14.60)
	Fatigue	8	154	8	170	1.11 (0.41 to 3.03)
	Hot flush	31	154	41	170	0.79 (0.47 to 1.34)
	BCS	69	154	60	170	1.49 (0.95 to 2.33)
Let *vs*. Ana	COR	95	127	85	123	1.33 (0.76 to 2.31)
	TC	121	127	114	123	1.59(0.55 to 4.61)
	Fatigue	10	127	9	123	1.08 (0.42 to 2.76)
	Hot flush	24	127	18	123	1.36 (0.70 to 2.65)
	BCS	11	127	24	123	**0.39 (0.18 to 0.84)**
Ana *vs*. Ana+Gef	COR	48	79	52	109	1.70 (0.94 to 3.05)
	TC	74	85	95	121	1.84 (0.85 to 3.97)
	Fatigue	8	85	5	121	2.41 (0.76 to 7.64)
	Hot flush	11	85	7	121	2.42 (0.90 to 6.53)
	BCS	27	85	51	121	0.64 (0.36 to 1.14)
Ana *vs*. Ana+Tam	COR	42	113	43	109	0.91 (0.53 to 1.56)
	TC	106	113	100	109	1.36 (0.49 to 3.80)
	Fatigue	6	113	8	109	0.71 (0.24 to 2.11)
	Hot flush	20	113	30	109	0.57 (0.30 to 1.07)
	BCS	21	113	11	109	2.03 (0.93 to 4.45)
	Outcome	Events	Total	Events	Total	OR (95% CI)
Ana *vs*. Tam	COR	123	276	99	259	1.30 (0.92 to 1.83)
	TC	308	341	302	331	0.89 (0.53 to 1.51)
	Fatigue	11	341	22	331	**0.47 (0.22 to 0.98)**
	Hot flush	39	341	44	331	0.84 (0.53 to 1.33)
	BCS	82	255	45	228	**1.95 (1.26 to 3.02)**
Ana *vs.* Exe (<20wks)	COR	85	123	78	124	1.32 (0.78 to 2.24)
	TC	114	123	114	124	1.11 (0.44 to 2.84)
	Fatigue	9	123	10	124	0.90 (0.35 to 2.30)
	Hot flush	18	123	10	124	1.95 (0.86 to 4.42)
	BCS	24	123	17	124	1.53 (0.77 to 3.01)
Tam *vs*. Tam+Ana	COR	39	108	43	109	0.87 (0.50 to 1.50)
	TC	101	108	100	109	1.30 (0.47 to 3.62)
	Fatigue	8	108	8	109	1.01 (0.36 to 2.80)
	Hot flush	28	108	30	109	0.92 (0.51 to 1.68)
	BCS	8	108	11	109	0.71 (0.27 to 1.85)
Exe (<20wks) *vs*. Exe (≥20wks)	COR	11	26	12	25	0.79 (0.26 to 2.40)
	TC	26	26	25	26	3.12 (0.12 to 80.12)
	Fatigue	–	26	–	25	–
	Hot flush	–	26	–	25	–
	BCS	4	26	1	25	4.36 (0.45 to 42.08)
Exe (<20wks) *vs*.Exe (<20wks)+Cel	COR	13	24	18	30	0.79 (0.27 to 2.33)
	TC	22	24	29	30	0.38 (0.03 to 4.46)
	Fatigue	1	24	3	30	0.39 (0.04 to 4.02)
	Hot flush	12	24	6	30	**4.00 (1.20 to 13.28)**
	BCS	–	–	–	–	–

CT = chemotherapy, Ana = anastrozole, Tam = tamoxifen, Gef = gefitinib, Let = letrozole, Exe(<20wks) = Exemestane (<20wks), Exe (≥20wks) = Exemestane (≥20wks), Cel = Celecoxib, Eve = Everolimus.

**Table 3:**
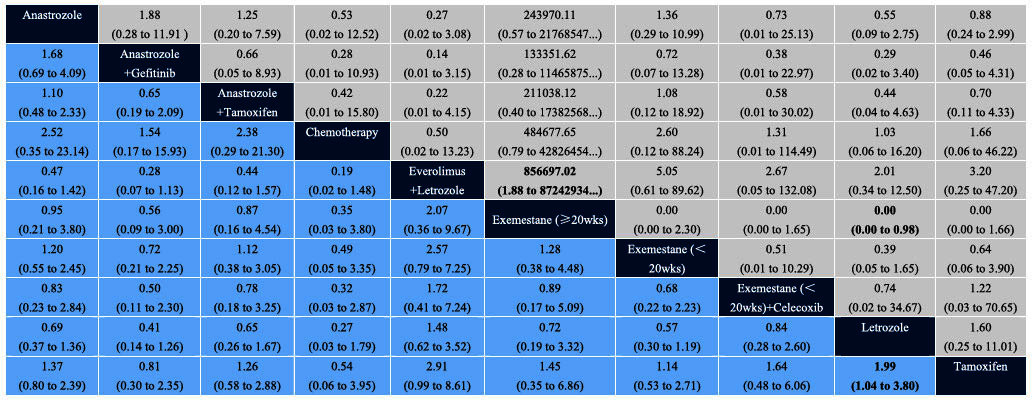
Indirect comparison of COR and TC.

Light blue boxes represent clinical objective response (COR, 95%CI), gray boxes represent treatment completion (TC, 95%CI). ORs in light blue boxes represent the column-defining treatment compared with row-defining treatment, and ORs in gray boxes represent the row-defining treatment compared with column-defining treatment. For COR, ORs greater than 1 favor the column-defining treatment. For TC, ORs greater than 1 favor the row-defining treatment.

**Table 4:**
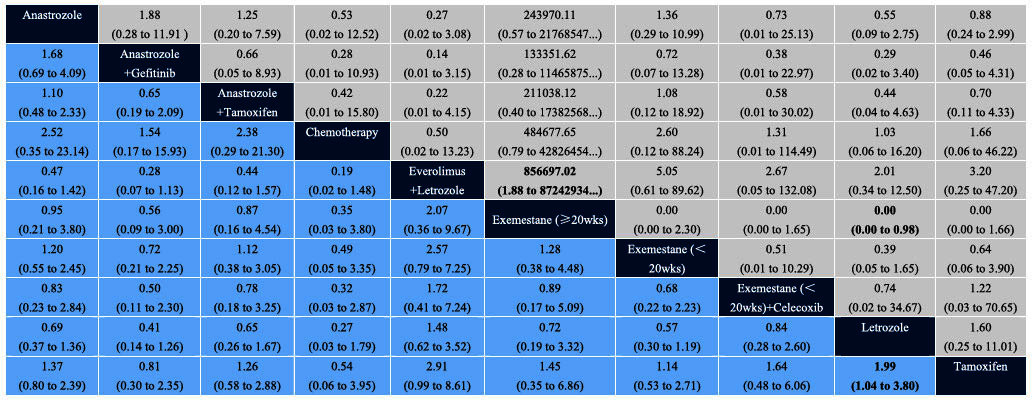
Indirect comparison of fatigue and hot flush.

Light blue boxes represent clinical fatigue (95%CI), gray boxes represent hot flush (95%CI). ORs in the light blue boxes represent the column-defining treatment compared with row-defining treatment, and ORs in gray boxes represent the row-defining treatment compared with column-defining treatment. For Fatigue, ORs greater than 1 favor the row-defining treatment, indicating that the incidence of fatigue is lower. For hot flush, ORs greater than 1 favor the column-defining treatment, indicating that the incidence of hot flush is lower.

**Table 5:** The first and second highest percentage within each intervention over ranks.

**Rank**	**Ana**	**Ana+Gef**	**Ana+Tam**	**CT**	**Eve+Let**	**Exe (≥20wks)**	**Exe (<20wks)**	**Exe (<20wks)+Cel**	**Let**	**Tam**
COR	4,6(20%) **5(29%)**	**9(36%)** 10(22%)	5(16%) 6(16%)	9(10%) **10(58%)**	**1(62%)** 2(20%)	1(14%) 2(13%)	**7(23%)** 8(19%)	2(16%) 3(18%)	**2(35%)** 3(33%)	7(22%) **8(28%)**
TC	5(22%) **6(25%)**	8(22%) **9(36%)**	7(16%) 8(19%)	1(22%) 2(18%)	**1(44%)** 2(30%)	9(2%) **10(93%)**	7(19%) **8(22%)**	1(22%) 9(17%)	**3(34%)** 4(21%)	4(19%) 5(20%)
PR	**4(21%)** 5(20%)	8(18%) **9(34%)**	3(16%) 4(14%)	9(13%) **10(70%)**	**1(45%)** 2(18%)	8(15%) 9(14%)	6(19%) **7(22%)**	8(17%) 9(16%)	**3(25%)** 4(17%)	**6(20%)** 7(19%)
CR	7,9(20%) **8(26%)**	4(15%) 3(18%)	9(18%) **10(34%)**	**1(94%)** 6(7%)	**4(20%)** 5(15%)	2(32%) 3(17%)	5(18%) 6(19%)	**2(34%)** 3(25%)	5(23%) **6(25%)**	**9(26%)** 10(23%)
BCS	**2(52%)** 3(26%)	**1(69%)** 2(14%)	5(18%) 6(22%)	7(30%) **8(55%)**	–	7(36%) 8(40%)	**3(29%)** 4(20%)	–	**4(30%)** 5(28%)	5(29%) **6(37%)**
Fatigue	6(22%) **7(26%)**	8(18%) **9(61%)**	2(19%) 3(15%)	**8(23%)** 9(18%)	**1(72%)** 2(14%)	–	6(19%) 7(21%)	2(18%) 3,4(11%)	3(23%) **4(26%)**	2(23%) **3(25%)**
Hot flush	3(27%) **4(31%)**	6(20%) **7(32%)**	**1(57%)** 2(16%)	8(33%) 9(43%)	**5(31%)** 6(32%)	–	**6(41%)** 7(29%)	8(42%) **9(47%)**	3(26%) 4(29%)	**2(37%)** 3(25%)
SMAA (COR, TC)	4(23%) **5(24%)**	8(22%) **9(26%)**	5–7(15%)	9(22%) 10(26%)	**1(64%)** 2(19%)	9(17%) **10(50%)**	6(18%) 7(20%)	2(16%) 3(17%)	**2(41%)** 3(31%)	6(19%) **7(22%)**

For fatigue and hot flush, rank 1 was worst and rank N was best. For others, rank 1 was best and rank N was worst. Bold figures represent the highest probabilities associated with the individual interventions and their associated ranks. Ana = anastrozole, Gef = gefitinib, Tam = tamoxifen, CT = chemotherapy, Eve = Everolimus, Let = letrozole, Exe (<20wks) = Exemestane (<20wks), Exe (≥20wks) = Exemestane (≥20wks), Cel = Celecoxib.
