# Supplementary material for: Network Meta-Analysis of the Effectiveness of Neoadjuvant Endocrine Therapy for Postmenopausal, HR–Positive Breast Cancer
Source: Sci Rep. 2016 May 13;6:25615. doi: 10.1038/srep25615 (PMC4865840; doi:10.1038/srep25615)
Supplement: Supplementary Information [file srep25615-s1.pdf]

Supplementary information

Network Meta-Analysis of The Effectiveness of Neoadjuvant Endocrine Therapy for postmenopausal, HR–Positive Breast Cancer

Wei Wang[1*], Chenghao Liu[*], Wenbin Zhou[1], Tiansong Xia[2] , Hui Xie[3] , Shui Wang[※]

Department of Breast Surgery, The First Affiliated Hospital with Nanjing Medical University, 300 Guangzhou Road, 210029 Nanjing, China

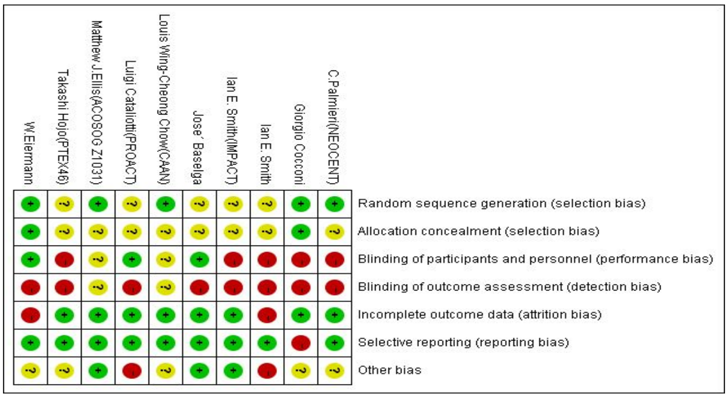

Supplementary Figure 1. Risk of bias tool, developed by the Cochrane Collaboration, to assess the potential limitations in randomized trials separates a judgment about risk of bias from a description of the support for that judgment, for a series of items covering different domains of bias.

"+ = low risk of bias; - = high risk of bias; ? = unclear risk of bias."

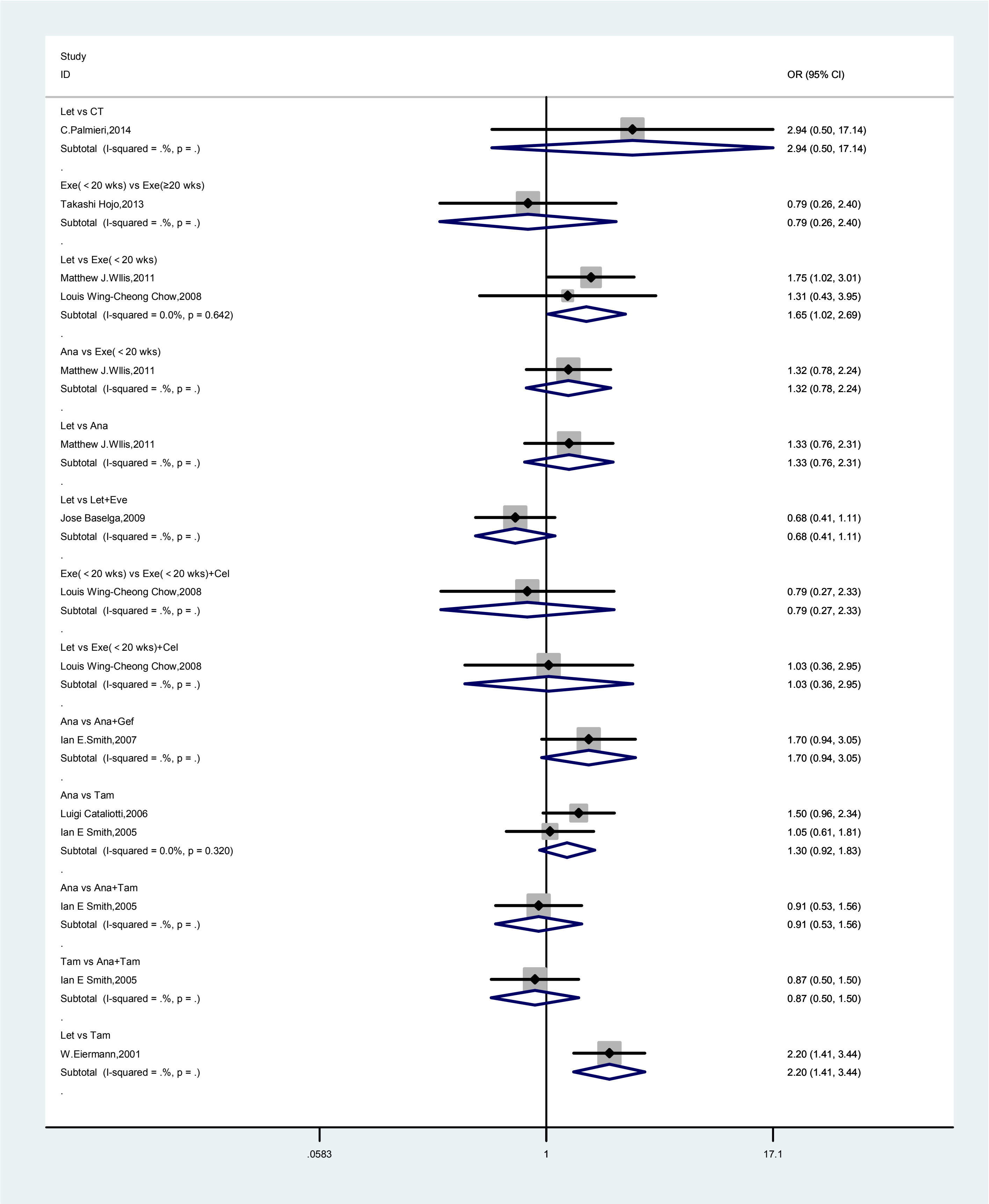

Supplementary Figure 2. Forest plots of COR

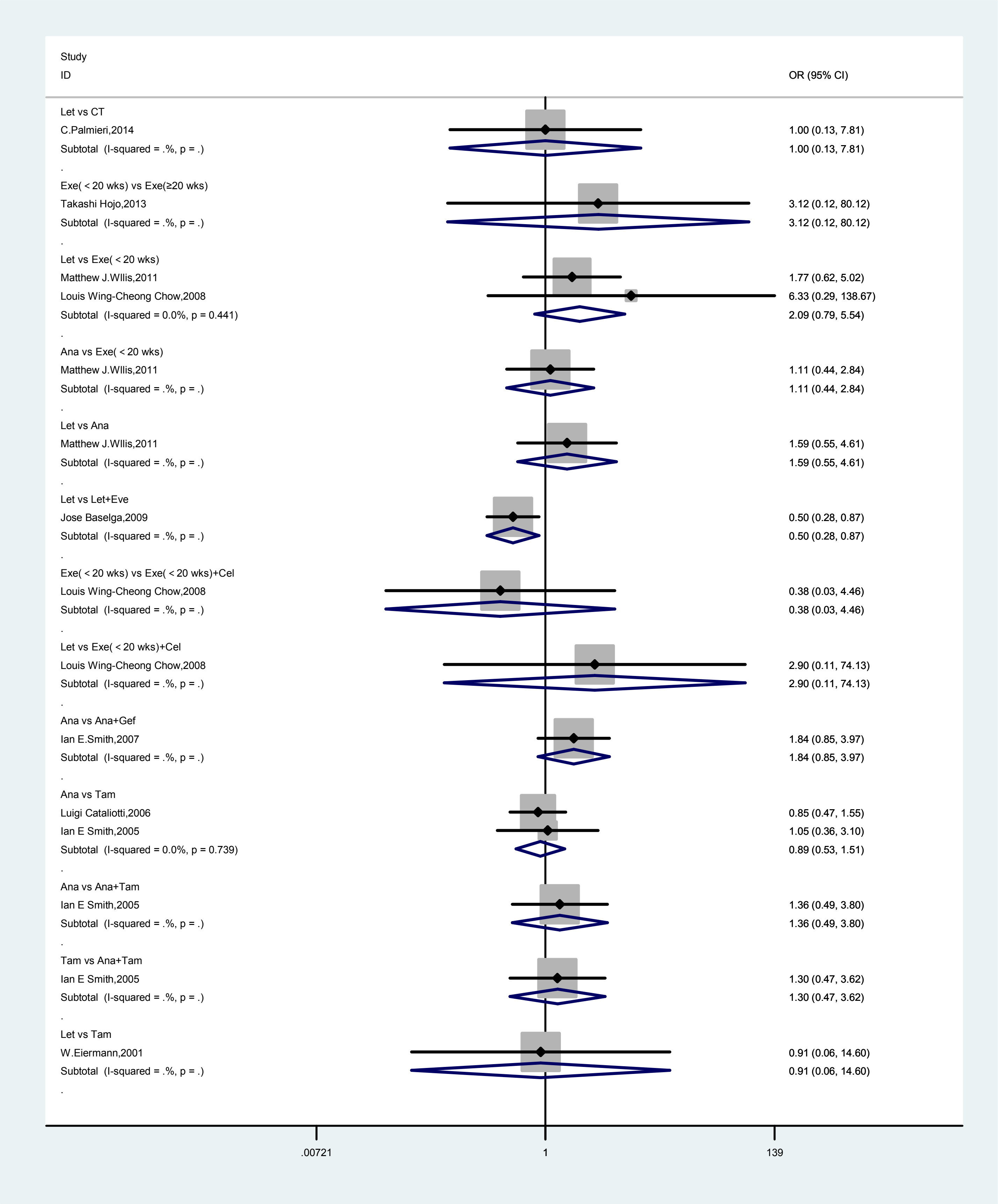

Supplementary Figure 3. Forest plots of TC

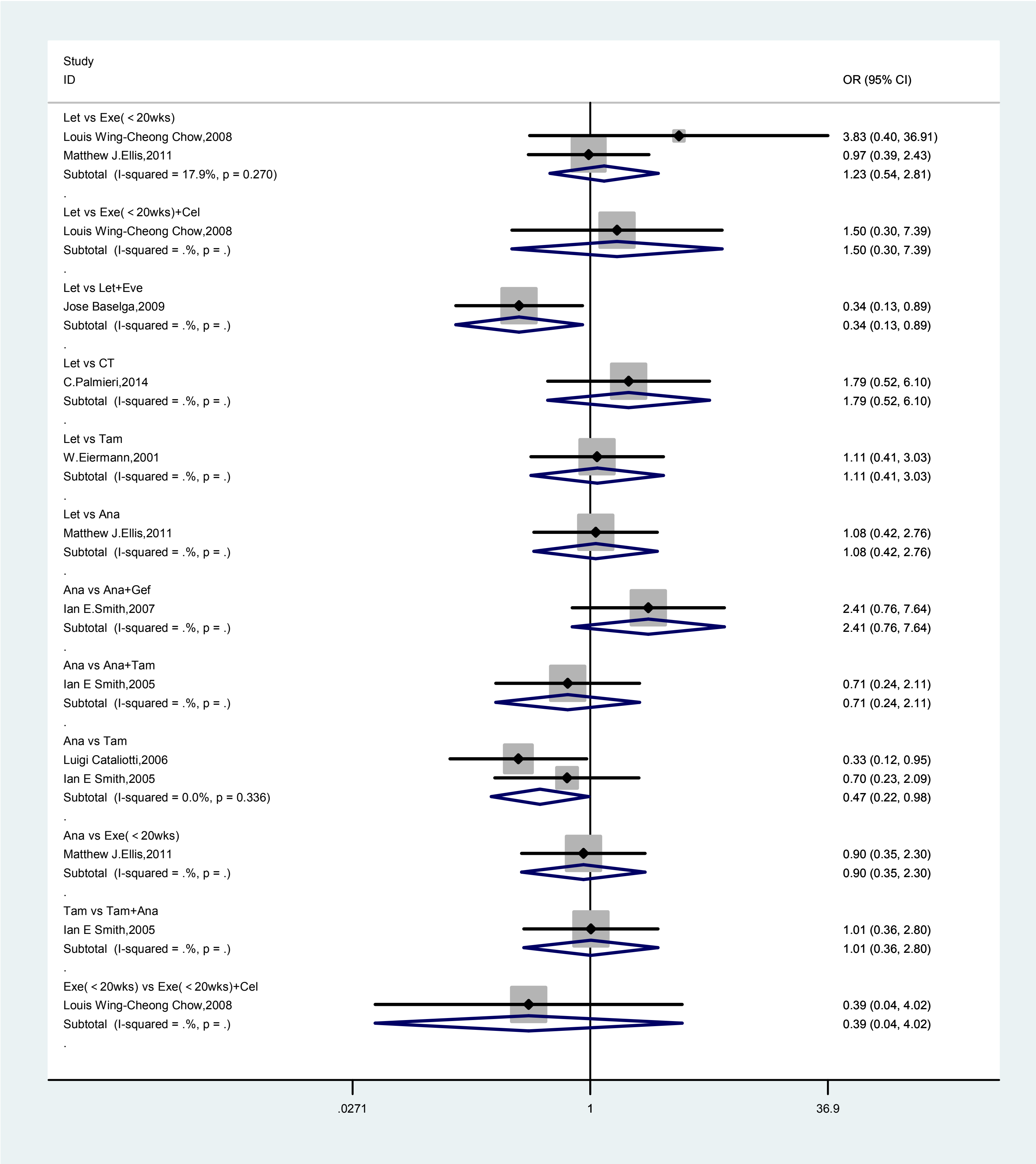

Supplementary Figure 4. Forest plots of fatigue

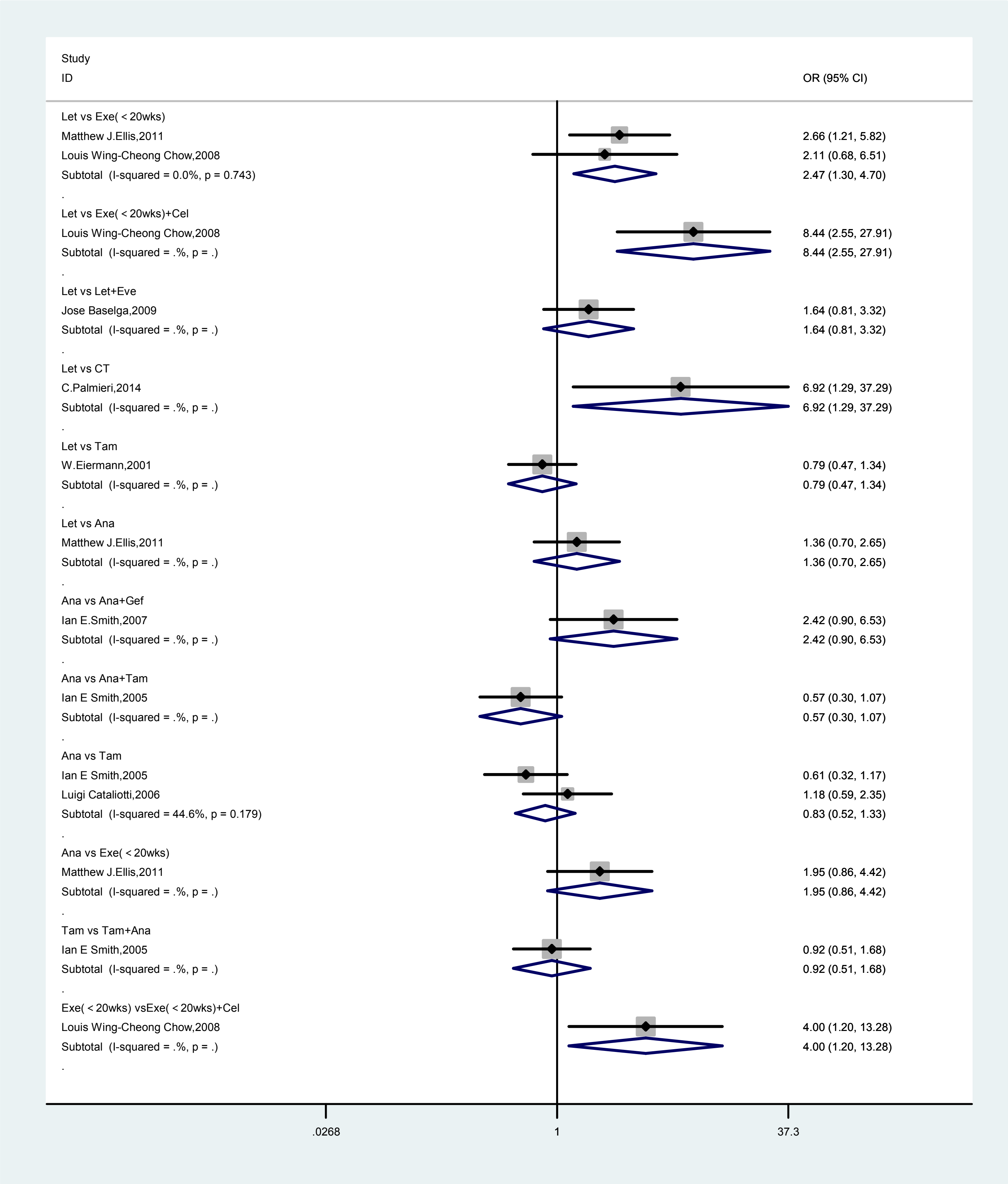

Supplementary Figure 5. Forest plots of hot flash

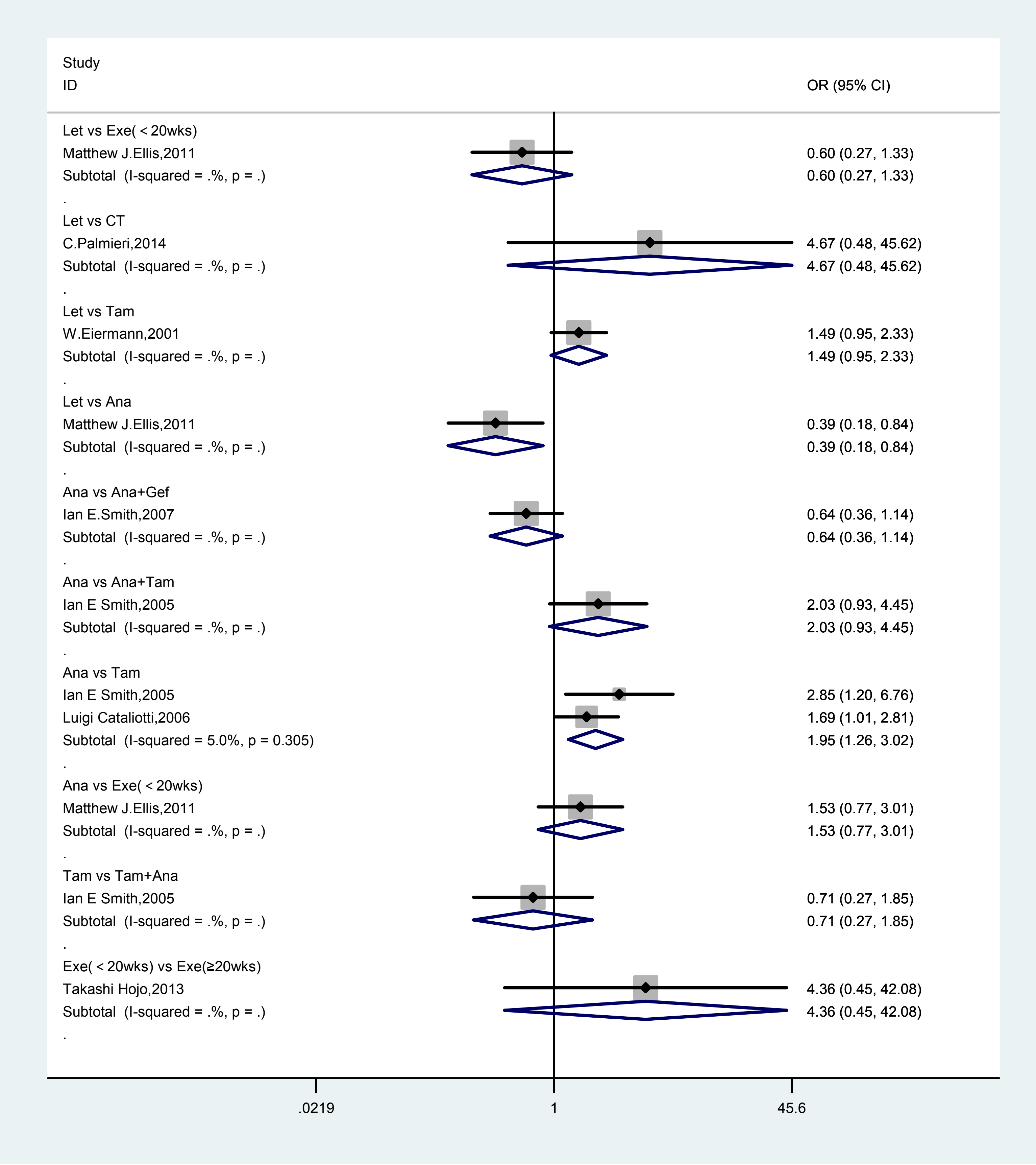

Supplementary Figure 6. Forest plots of BCS

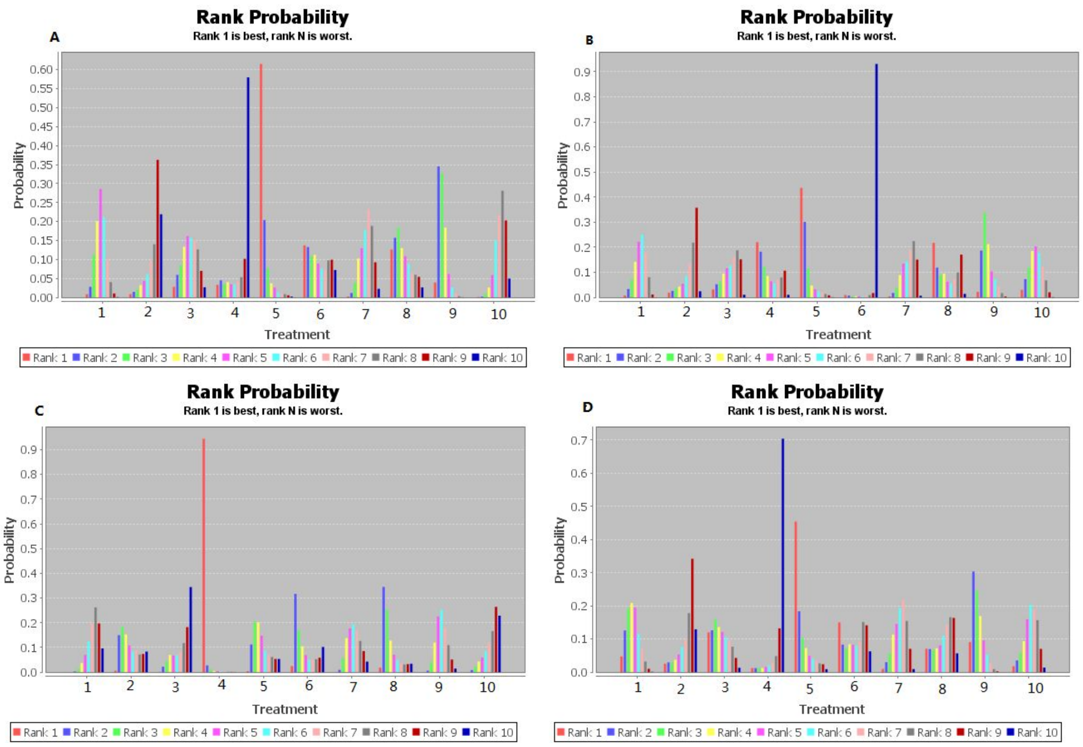

Supplementary Figure 7-10. Ranking for clinical objective response (A), treatment completion (B), complete response (C), partial response (D). Rank 1 represented best and rank N represented worst.

1=Anastrozole, 2=Anastrozole+Gefitinib, 3=Anastrozole+Tamoxifen, 4=chemotherapy, 5=Everolimus+Letrozole, 6=Exemestane (≥20wks), 7=Exemestane (＜20wks), 8=Exemestane (＜20wks)+Celecoxib, 9=Letrozole, 10=Tamoxifen

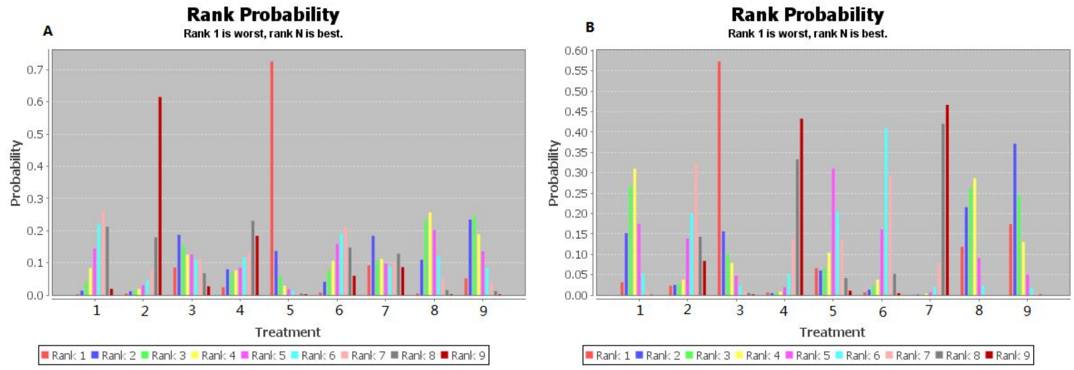

Supplementary Figure 11,12. Ranking for fatigue (A) and hot flash (B). Rank 1 represented worst and rank N represented best.

1=Anastrozole, 2=Anastrozole+Gefitinib, 3=Anastrozole+Tamoxifen, 4=chemotherapy, 5=Everolimus+Letrozole, 6=Exemestane (＜20wks), 7=Exemestane (＜20wks)+Celecoxib, 8=Letrozole, 9=Tamoxifen

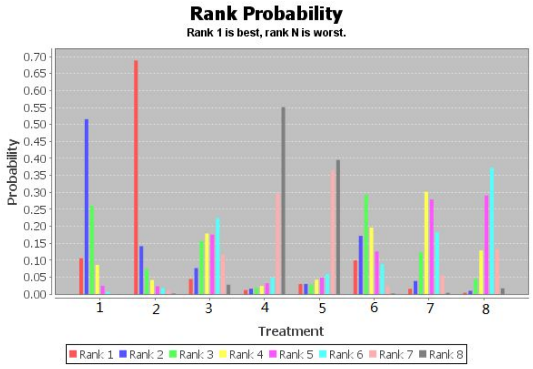

Supplementary Figure 13. Ranking for breast conserving surgery. Rank 1 represented best and rank N represented worst.

1=Anastrozole, 2=Anastrozole+Gefitinib, 3=Anastrozole+Tamoxifen, 4=chemotherapy, 5=Exemestane (≥20wks), 6=Exemestane (＜20wks),7=Letrozole, 8=Tamoxifen

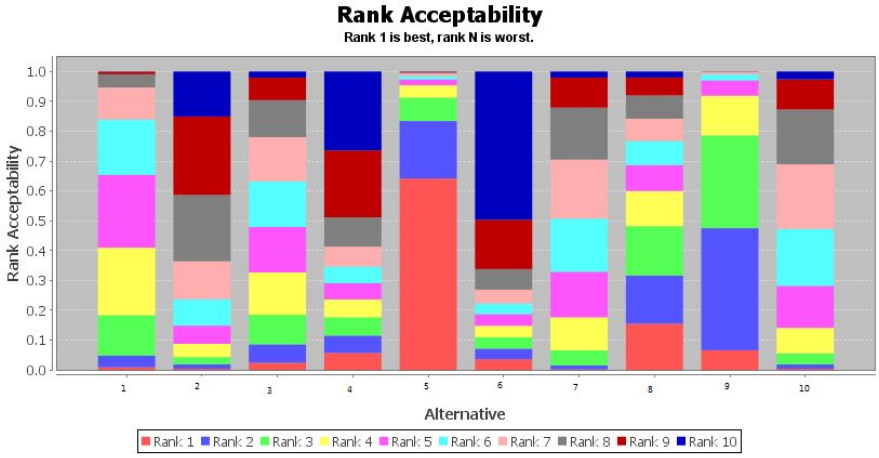

Supplementary Figure 14. Ranking for SMAA benefit-risk analysis based on synthesizing COR and TC. The bars indicate the probability that each treatment is the best, second best, etc. (Rank1 is best ,rank N is worst)

1=Anastrozole, 2=Anastrozole+Gefitinib, 3=Anastrozole+Tamoxifen, 4=CT, 5=Everolimus+Letrozole, 6=Exemestane (≥20wks), 7=Exemestane (＜20wks), 8=Exemestane (＜20wks)+Celecoxib, 9=Letrozole, 10=Tamoxifen
